# Systematic review of ketogenic diet use in adult patients with status epilepticus

**DOI:** 10.1002/epi4.12370

**Published:** 2019-11-24

**Authors:** Sherif Hanafy Mahmoud, Ethos Ho‐Huang, Jessica Buhler

**Affiliations:** ^1^ Faculty of Pharmacy and Pharmaceutical Sciences University of Alberta Edmonton AB Canada

**Keywords:** ketogenic diet, seizures, status epilepticus

## Abstract

Status epilepticus (SE) is a medical emergency that is associated with a significant morbidity and mortality. Recently, there has been significant interest in the use of ketogenic diets (KD) in the management of SE. KD is a high‐fat, low‐carbohydrate, and adequate protein diet that has been shown to be a safe and effective adjuvant to present SE management in patients with refractory epilepsy. Many case reports and case series have demonstrated the potential safety and effectiveness of KD for the acute treatment of SE; however, quality studies remain scarce on this topic. The purpose of this systematic review is to summarize the available evidence for the safety and effectiveness of KD in adults with SE. A literature search was performed in MEDLINE, EMBASE, Cochrane Library, and CINAHL (September 14, 2018). The search was repeated on March 27, 2019, to include any studies published since the original search. Keywords related to KD and SE were used. Studies were selected based on the reported use of the KD in SE. The search resulted in a total of 954 records. After screening and full‐text review, 17 articles were included in this review: four observational studies, 10 case reports, and 3 case series. Based on the observational studies, a total of 38 Patients with SE have been reported. KD was successful in achieving cessation of SE in 31 Patients (82%). The most common adverse effects reported were metabolic acidosis, hyperlipidemia, and hypoglycemia. The current limited evidence suggests that KD might be considered as an option for adult patients with SE. Although promising, the results need to be interpreted with caution due to the inherent bias, confounding and small sample size of the included studies. A randomized controlled trial is recommended to establish role of KD in the management of SE in adults.

**Key Points**

- A systematic review of the use of ketogenic diets (KD) in adults with status epilepticus
- A total of 17 articles were included: 1 prospective and 3 retrospective observational studies, 10 case reports, and 3 case series.
- A total of 38 Patients with SE have been reported in observational studies. KD was successful in achieving cessation of SE in 31 (82%).
- The most common adverse effects reported were metabolic acidosis, hyperlipidemia, and hypoglycemia.
- The results need to be interpreted with caution due to the inherent bias, confounding and small sample size of the included studies.

## 1 INTRODUCTION

Status epilepticus (SE) is a life‐threatening medical emergency where patients develop prolonged seizures exceeding 5 minutes in duration or multiple seizures with no return to the baseline in between.^1^ If SE fails to remit despite an initial benzodiazepine (BDZ) and another antiepileptic drug (AED), it is considered refractory status epilepticus (RSE).^2^ If seizure activity persists despite intravenous anesthetics or recurrence of seizure activity following the weaning of intravenous anesthetics, it is considered superrefractory status epilepticus (SRSE).^3^ RSE and SRSE are associated with a significant increase in morbidity and mortality compared to nonrefractory SE. While overall mortality of nonrefractory SE is high at 20%, the mortality rate rises significantly and has been reported anywhere between 23% and 57% in patients with RSE and SRSE.^2^, ^3^, ^4^ Therefore, determination of safe and effective therapies is essential.

Ketogenic diets (KD) are high‐fat, low‐carbohydrate, and adequate protein diets that are designed to mimic a fasting state and induce ketone body production through fat metabolism.^4^, ^5^ The ketones become an alternative source of energy for the brain and have shown to exhibit antiepileptogenic and neuroprotective properties.^6^, ^7^ The mechanism of action of the KD antiepileptogenic effects is largely unknown. However, several mechanisms have been postulated including enhanced ϒ‐aminobutyric acid (GABA) production, reduction of reactive oxygen species, as well as enhanced metabolic sensitivity of K_ATP_ channels.^8^ In addition, it has been suggested that KD has possible antiinflammatory properties that could be beneficial in the setting of epilepsy.^9^ Because of these effects, there has been great interest of KD in seizure management. The ketogenic antiepileptic diet was first introduced in 1921 and has been shown to be a safe and effective alternative in those with pharmacoresistant epilepsies, surgical contraindications, and medication intolerances.^10^ Due to the success in other etiologies, over the last years, there has been significant interest in the use of KD in the management of SE.^11^ Seizure control in SE has been achieved in diets that consist of a ratio of ketone‐producing fats to nonketogenic proteins and carbohydrates from 3:1‐4:1.^6^ It should be noted that these high‐ratio diets are the ones reported in acute care. Patients who are discharged on this diet may have the ratios adjusted to meet their personal requirements long term. Many case reports and case series have demonstrated the potential safety and effectiveness of KD for the acute treatment of SE; however, quality studies remain scarce on this topic.

**Figure 1:**
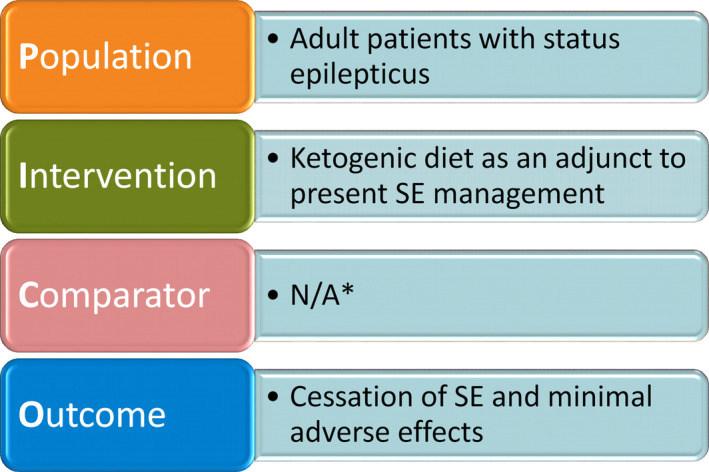
Focused clinical question in PICO format (Population, Intervention, Comparator, Outcome); *None of the reviewed studies included a comparison arm

Although current evidence suggests a role for KD in seizure management, more studies are required to truly assess the effectiveness and safety considerations when initiating therapy. Given the success of KD in terminating refractory and superrefractory cases of status epilepticus in pediatrics,^5^, ^7^ it is hypothesized that KD might be an effective treatment option for adult patients as well. From this, the following research question (Figure 1) was developed: In adult patients with SE, does the addition of KD as an adjunct to present SE management result in cessation of SE with minimal adverse effects? Therefore, the aim of this systematic review is to summarize the available evidence for the safety and effectiveness of KD in adult patients with SE. To our knowledge, this is the first systematic review summarizing the use of the KD in SE in adults.

## 2 METHODS

This review was conducted using a prespecified protocol and following the Preferred Reporting Items for Systematic Reviews and Meta‐Analyses (PRISMA) checklist.^12^

### 2.1 Search strategy

A literature search was performed in MEDLINE (1946 to September 14, 2018), EMBASE (1974 to September 14, 2018), Cochrane Library (1999 to September 14, 2018), and CINAHL (September 14, 2018). The search was repeated on March 27, 2019, to include any studies published since the original search. The following keywords were used: (“ketogenic” OR “ketosis” OR “keto diet” OR “ketoacid” OR ketogenesis”) AND (“status epilepticus” OR “long adj3 seizure” OR “continuous adj3 seizure” OR “unremitting adj3 seizure” OR “nonconvulsive adj3 seizure” OR “Kojevnikov epileps” OR “Kojevnikov syndrome” OR “Kozhevnikov epileps” OR “Kozhevnikov syndrome” OR “generalized convulsive SE” OR “petit mal status” OR “absence status” OR “subtle SE” OR “nonconvulsive SE” OR “absence SE” OR “complex partial SE” OR “simple partial SE” OR “prolong adj3 seizure”). The keywords used in the study were chosen in conjunction with a library information specialist to ensure a comprehensive search that covered variations in the KD and condition subtypes. The reference lists of the included articles were also searched manually to find additional eligible articles. Search was undertaken by SHM and EH.

### 2.2 Study selection

**Figure 2:**
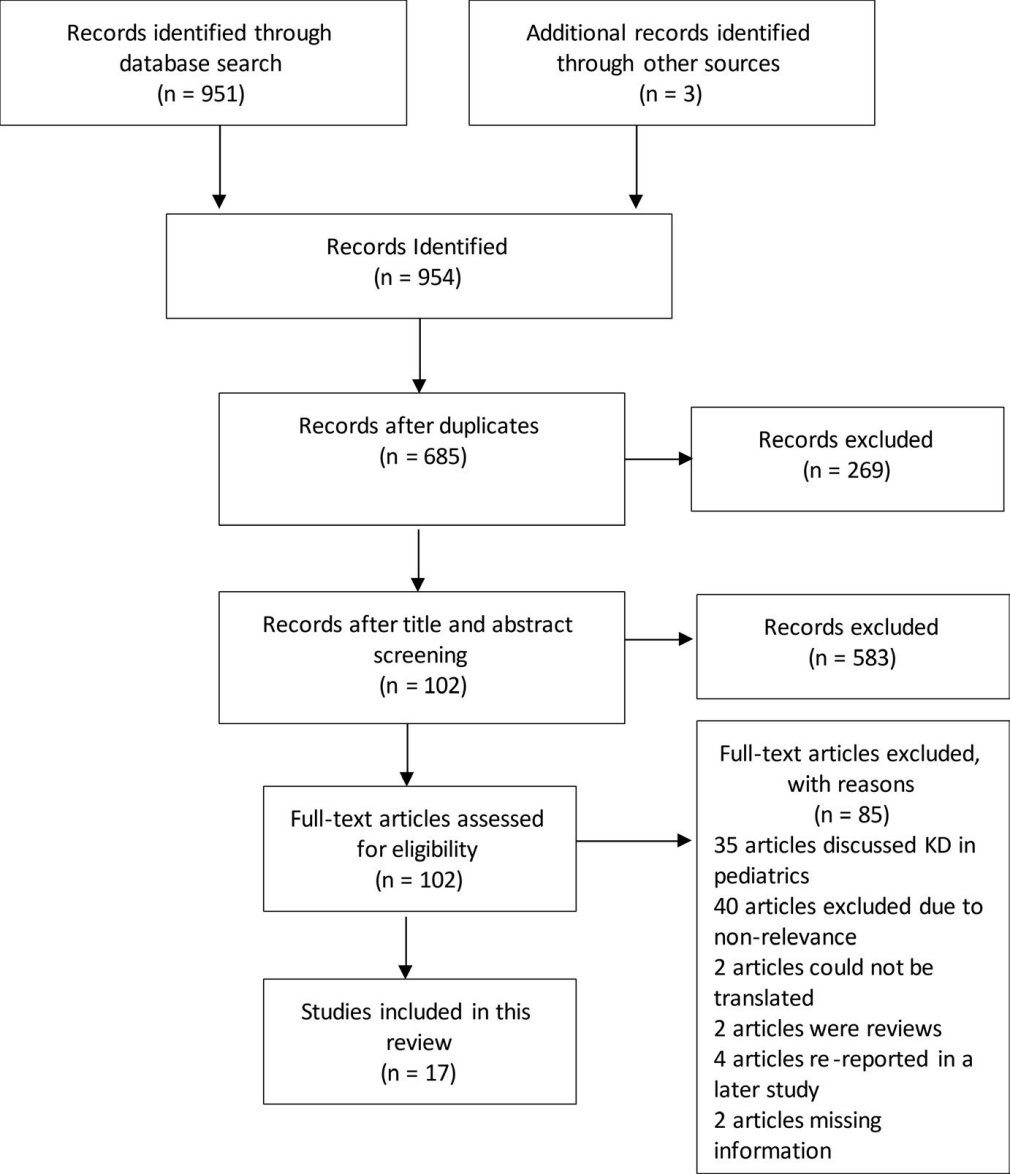
Flow diagram of the search strategy and results

Studies were selected based on the reported use of the KD in adult patients with SE. All study designs and outcomes related to cessation of SE were included. Nonrelevant studies, non–English‐language studies that could not be translated using Google Translate, nonhuman studies, commentaries, opinion articles, editorials, and review articles were excluded after title and abstract screening. The full‐text screening of the remaining articles was conducted to determine their eligibility for inclusion in the systematic review as depicted in Figure 2. Study selection was undertaken by SHM and EH. In case of any discrepancies between the authors, further discussion was done to reach a consensus.

### 2.3 Data collection and quality assessment

Data collected included year of publication, study type, number of participants treated with the KD, and participants’ age and sex, previous history of seizures and epilepsy, and SE type. When available, KD ratio (fats: carbohydrate, proteins), etiology, reported side effects, order of initiation, time from SE onset to KD initiation, time to ketosis, and time to SE control in KD responders were also collected. Outcome data collected included KD success in resolving SE, posttreatment information, and adverse reactions attributed to KD use. Individual articles were critically appraised using Joanna Briggs Institute (JBI) Appraisal Checklists. The appropriate checklist was selected based on the study design.^13^ The overall level of evidence was graded using the Grading of Recommendations, Assessment, Development and Evaluation (GRADE) working group criteria.^14^ Data extraction and quality assessment were undertaken by all authors.

## 3 RESULTS

As depicted in Figure 2, the search resulted in a total of 954 records. Of these, 656 came from EMBASE, 190 from MEDLINE, 45 from the Cochrane Library, 60 from CINAHL, and three from manual search. After screening, full‐text review, and applying the exclusion criteria, 17 articles were included in this review (Tables 1 and 2): 1 prospective and 3 retrospective observational studies, 10 case reports, and 3 case series.

**Table 1:** Summary of observational studies regarding ketogenic diet effectiveness in status epilepticus in adults

Author (year)	Study design	n	Male n (%)	Age (years) Median (IQR)	History of seizures/ Epilepsy n (%)	KD Type	Etiology (n)	No. of AEDs tried before initiation^c^ Median (IQR)	Time from SE onset to KD initiation (days)	Time to ketosis (days) Median (IQR)	Time to SE control in KD responders (days)	Resolution of SE n (%)	AEs (n)
Cervenka et al (2017)^4^	Prospective (SRSE)	15	5 (33)	47 (30)	6 (40)	4:1	NORSE (5) LGS (2) Anoxic brain injury (2) ICH (1) Encephalitis (1) Hemorrhagic infarct (1) Traumatic ICH (1) Epilepsy secondary to shaken baby syndrome (1) GBM (1)	8 (7)	10 (IQR 7)	2 (1)^a^ (100% ketosis)	5 (IQR 3)	11 (73)	Hyponatremia (1) Constipation (2) Metabolic acidosis (4) Hyperlipidemia (2) Hypoglycemia (1) Weight loss (1)
Francis et al (2018)^15^	Retrospective (RSE)	11	6 (55)	46 (41)	5 (41)	3:1 or 4:1	TBI (3) Anoxic brain injury (2) ICH (2) Ischemic stroke (1) AED nonadherence (1) EtOH withdrawal (1) Anti‐NMDA R encephalitis (1)	3 (1)	1 (IQR 2)	1 (2)^b^ (90.9% ketosis)	NR	8 (73)	Metabolic acidosis (8) Hypoglycemia (2) Hyponatremia (1) Elevated LFTs (1)
Park et al (2019)^17^	Retrospective (SRSE)	2	1 (50)	21;40	NR	NR	FIRES (2)	NR	12;37	NR	At 7 days 1 seizure free; 1 > 50% seizure reduction	2 (100)	Nausea; vomiting (1)
Thakur et al (2014)^16^	Retrospective (Abstract) (SRSE)	10	4 (40)	33 (21)	NR	3:1 (1) 4:1 (9)	Encephalitis (7) Cortical dysplasia (1) Anoxic brain injury (1) Neurocysticercosis/subtherapeutic AEDs (1)	7 (7)	21.5 (IQR 28)	3 (5) (90% ketosis)	3 (IQR 8)	10 (100)	Metabolic acidosis (3) Hypertriglyceridemia (3)

Abbreviations: AE, adverse events; FIRES, febrile‐induced refractory epilepsy syndrome; GBM, glioblastoma multiforme; ICH, intracerebral hemorrhage; IQR, interquartile range; LFTs, liver function tests; LGS, Lennox‐Gastaut syndrome; NMDA R, N‐methyl D‐aspartate receptor; NORSE, new‐onset refractory status epilepticus; NR, not reported; TBI, traumatic brain injury.

a Ketosis defined as urine acetoacetate >40 mg/dL and/or serum ‐hydroxybutyrate ≥2 mmol/L.

b Defined as detection of urine ketones

c Not including BDZ and anesthetics.

**Table 2:** Summary of case series and case reports regarding ketogenic diet effectiveness in adults with superrefractory status epilepticus

Author (year)	Sex	Age (years)	History of seizures/Epilepsy n (%)	SE Type (Seizure type)	KD Type	Etiology (n)	No. of AEDs tried before initiation^c^	Time from SE onset to KD initiation (days)	Time to ketosis (days)	Time to SE control in KD responders (days)	Resolution of SE	AEs (n)
Wusthoff et al (2010)^36^	F	29	Yes	SRSE (NCSE)	4:1	Rasmussen's encephalitis	7	101	10	4	Yes	NR
	M	34	No	SRSE (NCSE)	4:1	Viral encephalitis	5	20	8	6	Yes	NR
Blunck et al (2018)^26^	F	42	Yes	SRSE (NR)	4:1	AED switch	6	84	23 (7 d after starting dapagliflozin)	N/A	No	NR
Amer et al (2015)^25^	F	21	NR	SRSE (NR)	4:1	Anti‐NMDA R encephalitis	5	21	NR	14	Yes	NR
Martikainen et al (2012)^30^	F	26	No	RSE (NCSE)	LGIT	POLG‐related mitochondrial epilepsy	3	3	NR	4	Yes	NR
Strzelcyk et al (2013)^33^	F	21	Yes	SRSE (GCSE)	4:1	Lafora disease	6	16	3.5	NR	Yes	NR
Uchida et al (2017)^35^	F	20	NR	SRSE (NR)	NR	Anti‐NMDA R encephalitis	NR	NR	NR	NR	Yes	NR
Bodenant et al (2008)^6^ (French)	M	54	Yes	RSE (FSE)	4:1	Recent AED switch; pneumonia	7	31	NR	4	Yes	NR
Matsuzono et al (2014)^31^	M	22	No	SRSE (NCSE)	NR	Acute encephalitis with refractory repetitive focal seizures	11	155	NR	25	Yes	NR
Hakimi et al (2014)^28^ (Abstract)	F	44	No	SRSE (FSE)	NR	Creutzfeldt‐Jakob disease	5	27	NR	5	Yes	NR
Cash et al (2015)^27^ (Abstract)	M	NR (adult)	NR	RSE (Myoclonic SE)	4:1	Hypoxic‐ischemic injury	NR	22	NR^b^	NR	Yes	NR
Owusu et al (2017)^32^ (Abstract)	F	29	No	SRSE (NR)	NR	NORSE	16	27	37	NR	Yes	NR
Lin et al (2012)^29^ (Abstract)	F	19	NR	SRSE (NR)	NR	Anti‐NMDA R encephalitis	NR	NR	NR	14	Yes	NR
	M	49	NR	SRSE (NR)	NR	Encephalitis (unknown etiology)	NR	NR	NR	14	Yes	NR
Su et al (2015)^34^ (Abstract)	M	28	NR	RSE (NR)	NR	NR	NR	NR	NR^a^	NR	Yes n = 2 No n = 1	Elevated LFTs (n = 3) Hypertriglyceridemia (n = 2)
	F	58	NR	RSE (NR)	NR	NR	NR	NR		NR		
	F	31	NR	RSE (NR)	NR	NR	NR	NR		NR		

Abbreviations: AEDs, antiepileptic drugs; FSE, focal status epilepticus; GCSE, generalized convulsive status epilepticus; LFTs, liver function tests; LGIT, low‐glycemic index treatment; NCSE, nonconvulsive status epilepticus; NMDA R, N‐methyl D‐aspartate receptor; NORSE, new‐onset refractory status epilepticus; NR, not reported; POLG, mitochondrial polymerase gamma.

a 2 patients were able to achieve ketosis based on urine ketones and/or serum beta‐hydroxybutyrate levels (BOH).

b Ketosis indicated by blood ketone levels between 1.3 and 3.2 mmol/L

c Not including BDZ and anesthetics.

Appraisal of individual studies is shown in Table 3. Overall, the 4 observational studies were well reported and scored 70% or above using the JBI Checklists. Although well reported, the observational nature of the studies still results in low‐quality evidence according to the GRADE working group criteria. The case reports and case series were all graded as very low‐quality evidence according to the GRADE working group criteria.

**Table 3:** Appraisal of individual studies included in this review

Author (year)	A	B	C	D	E	F	G	H	I	J	K	Total
I. Critical Appraisal of Prospective Observational Studies (Cohort)												
Cervenka et al (2017)^4^	N/A	Y	Y	Y	N	Y	Y	Y	N/A	N/A	Y	7/8
II. Critical Appraisal of Retrospective Observational Studies												
Francis et al (2018) 15	Y	Y	Y	Y	Y	Y	Y	Y	Y	Y		10/10
Park et al (2019)^17^	N	Y	Y	Y	U	N	Y	Y	Y	Y		7/10
Thakur et al (2014) 16	Y	Y	Y	Y	U	Y	Y	Y	Y	Y		9/10
III. Critical Appraisal of Case Series												
Wusthoff et al (2010)^36^	Y	Y	Y	N	N	Y	Y	Y	Y			7/9
Lin et al (2012) (Abstract)^29^	U*	Y	Y	Y	Y	Y	U*	U*	U*			5/9
Su et al (2015)^34^ (Abstract)	Y	Y	Y	N	N	Y	Y	Y	U*			6/9
IV. Critical Appraisal of Case Reports												
Blunck et al (2018)^26^	Y	Y	Y	Y	Y	Y	N	Y				7/8
Amer et al (2015)^25^	Y	N	Y	Y	Y	N	N	Y				5/8
Martikainen et al (2012)^30^	Y	Y	Y	Y	Y	Y	Y	Y				8/8
Strzelcyk et al (2013)^33^	Y	Y	Y	Y	Y	Y	N	Y				7/8
Uchida et al (2017)^35^	Y	N	Y	Y	Y	N	N	Y				5/8
Bodenant et al (2008)^6^ (French)	Y	Y	Y	Y	Y	Y	N	Y				7/8
Matsuzono et al (2014)^31^	Y	Y	Y	Y	Y	Y	N	Y				7/8
Hakimi et al (2014)^28^ (Abstract)	Y	U*	Y	Y	U*	Y	U*	Y				5/8
Cash et al (2015)^27^ (Abstract)	U*	U*	Y	Y	U*	Y	U*	Y				4/8
Owusu et al (2017)^32^ (Abstract)	U*	U*	Y	Y	Y	U*	U*	Y				4/8

Note

*Appraisal was conducted using Joanna Briggs Institute Appraisal Checklists. ^13^ I Prospective Observational Studies*: A—Were the two groups similar and recruited from the same population? B—Were the exposures measured similarly to assign people to both exposed and unexposed groups? C—Was the exposure measured in a valid and reliable way? D—Were confounding factors identified? E—Were strategies to deal with confounding factors stated? F—Were the groups/participants free of the outcome at the start of the study (or at the moment of exposure)? G—Were the outcomes measured in a valid and reliable way? H—Was the follow‐up time reported and sufficient to be long enough for outcomes to occur? I—Was follow‐up complete, and if not, were the reasons to loss to follow‐up described and explored? J—Were strategies to address incomplete follow‐up utilized? K—Was appropriate statistical analysis used?

*II and III Case Series and Retrospective Observational Studies*: A—Were there clear criteria for inclusion in the case series? B—Was the condition measured in a standard, reliable way for all participants included in the case series? C—Were valid methods used for identification of the condition for all participants included in the case series? D—Did the case series have consecutive inclusion of participants? E—Did the case series have complete inclusion of participants? F—Was there clear reporting of the demographics of the participants in the study? G—Was there clear reporting of clinical information of the participants? H—Were the outcomes or follow‐up results of cases clearly reported? I—Was there clear reporting of the presenting site(s)/clinic(s) demographic information? J—Was statistical analysis appropriate? U* = Unsure as only abstracts available for analysis; U = Unsure; N/A = not applicable

*IV Case* *Reports*: A—Were the patient's demographic characteristics clearly described? B—Was the patient's history clearly described and presented as a timeline? C—Was the current clinical condition of the patient on presentation clearly described? D—Were diagnostic tests or assessment methods and the results clearly described? E—Was the intervention(s) or treatment procedure(s) clearly described? F—Was the postintervention clinical condition clearly described? G—Were adverse events (harms) or unanticipated events identified and described? H—Does the case report provide takeaway lessons? U* = Unsure (only abstracts available for analysis)

## 4 DISCUSSION

### 4.1 Effectiveness of KD in adults with SE

KD use in adult patients with superrefractory status epilepticus (SRSE) has been reported in a single prospective multicenter observational study.^4^ KD was initiated in 15 adult patients with SRSE with varied etiologies (Table 1). The primary outcome measure was the development of ketosis defined as urine acetoacetate ≥40 mg/dL and/or serum β‐hydroxybutyrate ≥2 mmol/L. Other outcomes included resolution of SRSE and Glasgow Coma Scale (GCS) and modified Rankin Scale (mRS) at discharge. One patient was withdrawn from the study following KD initiation. The number of AEDs used before KD initiation ranged from 5 to 12, and the time from SE onset to KD initiation ranged from 2 to 39 days. Ketosis was achieved in all patients with median time to ketosis of 2 days (IQR 1). KD was successful in achieving SRSE resolution in 11 Patients (73%). The authors concluded that KD might be a safe and effective option in adult patients with SRSE. Francis et al have reported similar rates of effectiveness (73%) in a retrospective study of 11 adult patients with RSE.^15^ However, in this study, KD was initiated early throughout the disease course (time from SE onset to KD initiation ranged from 0 to 3 days) suggesting possible effectiveness of KD early on in the disease course. Similarly, Thakur et al have reported a successful use of KD in 10 adult patients with SRSE. KD was successful in achieving cessation of SE in all the patients.^16^ In addition, a retrospective study by Park et al reporting a mixed cohort (adults and pediatrics) of patients treated with KD has reported two adult patients who were successfully treated with KD.^17^

Taken together, based on the observational studies described above, a total of 38 Patients with RSE/SRSE have been reported. KD was successful in achieving cessation of SE in 31 Patients (82%), suggesting a potential role of KD in the management of SE. The rates of KD reported in adults were similar to those reported in pediatric SE. Nine retrospective studies specifically looked at the effectiveness of KD in pediatrics for SE.^5^, ^11^, ^18^, ^19^, ^20^, ^21^, ^22^, ^23^, ^24^ A total of 85 Patients, 36 (42%) were males, were put on the KD for the treatment of RSE and SRSE, and it was effective in 64 (75%).

In addition to the above‐mentioned studies, there were 13 case reports and series that reported KD use in a total of 17 adult patients, six of those reported male.^6^, ^25^, ^26^, ^27^, ^28^, ^29^, ^30^, ^31^, ^32^, ^33^, ^34^, ^35^, ^36^ The age of patients ranged from 19 to 58 years. There were four Patients with a reported history of seizures/epilepsy. The number of reported AEDs tried before initiating KD, not including anesthetics and immunomodulators, ranged from 3 to 16. The reported time from SE onset to KD initiation ranged from 3 to 155 days, number of days to achieve ketosis ranged from 3.5 to 37 days, and the time to SE resolution ranged from 4 to 25 days. The KD was successful in 14 Patients for the treatment of RSE and SRSE (82%). It is worth noting that the patient in the case report by Uchida et al achieved resolution of SRSE with both KD and stiripentol.^35^

Although promising, the results need to be interpreted with caution due to the inherent bias, confounding and small sample size of the included studies. A multicenter randomized controlled trial is recommended to determine the role of KD in the management of SE in adults.

### 4.2 Safety of KD in adults with SE

Safety of KD diet in critically ill patients with SE is another important aspect to consider. In patients with refractory epilepsy, KD is generally well tolerated with reported adverse effects such as gastrointestinal symptoms, metabolic acidosis, nephrolithiasis, and increased propensity to infections.^37^ Five of the studies used in this review reported adverse effects from KD.^4^, ^15^, ^16^, ^17^, ^34^ The most common adverse effects reported in the reviewed studies were metabolic acidosis, hyperlipidemia, and hypoglycemia. Metabolic acidosis in some patients was reported to be persistent despite administration of bicarbonate therapy and resulting in KD discontinuation. This suggests the need for careful monitoring of adverse effects when KD is being utilized. It should be noted that none of the studies which reported adverse effects contained defining parameters for each of the stated adverse effects.

One important additional safety aspect of KD was the possible interaction between KD and propofol. Concomitant administration of KD and propofol might potentially increase the risk of propofol infusion syndrome. A case of fatal propofol infusion syndrome in 10 years old boy has been reported in the literature.^38^ Although there is no sufficient evidence to support or refute this possible interaction, the cumulative effects of propofol and KD on metabolic derangements (altered fatty acid oxidation) suggest avoiding propofol in patients treated with KD. Propofol therapy was one of the main reasons for the reported delay in KD initiation.

### 4.3 KD administration protocols in adults with SE

KD ratio of 4:1 of fats to nonketogenic proteins and carbohydrates was the most commonly reported regimen in adult patients (Tables 1 and 2). In contrast, pediatric SE studies have reported other regimens in addition to the 4:1 ratio such as 3:1^18^, ^20^, ^21^, ^24^, ^39^, 5:1^5^, ^18^, 6:1^40^, 1:1, 2:1, 2.75:1, 3.2:1, 3.5:1, and 4.5:1^18^, ^20^, ^21^, ^37^. In addition, less restrictive forms of the KD have been used to successfully induce ketone body production, such as the modified Atkins diet (MAD) in Kumada et al and a low‐glycemic index diet (LGIT) in Martikainen et al^30^, ^41^. A challenging aspect of KD therapy is the need to achieve ketosis to produce antiepileptogenic ketone bodies. In the reported studies, it can take up to 37 days to achieve sustained ketosis. The presence of carbohydrates that are commonly found in medications and intravenous fluids can potentially prolong the time for ketone body production. Obtaining a pharmacist consult to limit carbohydrates in fluids and administered medications is essential.

Two studies have reported the detailed standardized KD treatment protocols (Table 4).^4^, ^15^ Baseline steps, contraindications, KD regimen administration, and monitoring were very comparable. The median time to ketosis in both studies was 1‐2 days, with ketosis achieved in 25/26 Patients (96%) suggesting the potential success of these standardized protocols as opposed to earlier case reports where time to ketosis was at least 3.5 days.

**Table 4:** KD treatment protocol reported in the literature

Protocol	Details	Cervenka et al^4^	Francis et al^15^
At Baseline	Fasting lipid profile	**✓**	**✓**
	Urine ketones	**✓**	**✓**
	Comprehensive metabolic panel (CMP)	**✓**	**✓**
	Pregnancy test	**✓**	**✓**
	Continuous EEG	**✓**	**✓**
	Consult dietitian	**✓**	**✓**
	Consult pharmacist (to limit carbohydrates in fluids and administered medications)	**✓**	**✓**
	Vital; height and weight		**✓**
	CBC; selenium; vitamin D; amylase; lipase	**✓**	
Exclusion criteria (contraindications)	On propofol within 24 h	**✓**	**✓**
	Hemodynamic instability	**✓**	**✓**
	Pregnancy	**✓**	**✓**
	Liver failure	**✓**	**✓**
	Hypoglycemia (glucose <50 mg/dL)	**✓**	**✓**
	Ileus or any limited oral intake	**✓**	**✓**
	Fatty oxidation disorder or pyruvate carboxylate deficiency	**✓**	**✓**
	Hyponatremia; hypernatremia; hypocalcemia or pH < 7.2 within 24 h; cholesterol >300 mg/dL	**✓**	
KD formula	4:1 KD liquid formula	**✓**	**✓**
KD administration	Propofol discontinued × 24 h before KD initiation	**✓**	**✓**
	To start at ½ caloric intake for 24 h then increase to full intake	**✓**	
	NPO × 24 h		**✓**
Monitoring while on KD in the ICU	Glucose every 4 h (treat hypoglycemia <50 mg/dL, if present)	**✓**	**✓**
	Urine ketones q 24 h		**✓**
	Urine ketones q1 2 h	**✓**	
	Serum β‐hydroxybutyrate every 12 h	**✓**	
	Comprehesive metabolic panel within 48 h	**✓**	
Additional intervention	Administer vitamin D, multivitamin, and calcium	**✓**	**✓**
	Administer levocarnitine		**✓**

### 4.4 Limitations

This review is limited by the quality of the included studies. The findings should be interpreted with caution as the present evidence was merely based on observational studies, case series, and case reports. Case reports and case series have a strong bias toward only reporting positive outcomes. Additionally, there is a bias of attributing SE resolution to the last treatment tried when prior administered treatments, such as intravenous immunoglobulin (IVIG) or corticosteroids, may have produced a delayed response. Therefore, it is not clear if the reported KD effectiveness is attributed to KD or due to the concomitant management. In addition, there was considerable heterogeneity in patient characteristics, SE etiologies, definition of effectiveness, and time from SE onset to KD initiation. Furthermore, the definition of ketosis varied among studies; however, most of the reports agreed that persistence of urinary ketones is an indication of ketosis. As seen in Tables 1 and 2, SE etiologies have varied, and it seems that there is no sufficient sample size to associate effectiveness of KD in cessation of SE to a certain SE etiology. There were no randomized controlled trials identified that specifically looked at the KD in treating acute SE which greatly decreases the strength of evidence. Based on the critical analysis of the included evidence, the current evidence is not sufficient enough to conduct a metaanalysis and produce a point estimate.

## 5 CONCLUSION

The current evidence suggests that KD might be considered as an option for adult patients with SE especially in those with SRSE. A multicenter randomized controlled trial is recommended to establish role of KD in the management of SE in adults.

## DISCLOSURE

None of the authors has any conflict of interest to disclose.
